# Mast Cell Tryptase Contributes to Pancreatic Cancer Growth through Promoting Angiogenesis via Activation of Angiopoietin-1

**DOI:** 10.3390/ijms17060834

**Published:** 2016-05-27

**Authors:** Xiangjie Guo, Liqin Zhai, Ruobing Xue, Jieru Shi, Qiang Zeng, Cairong Gao

**Affiliations:** 1 Department of Forensic Medicine, Shanxi Medical University, 56 South Xinjian Road, Taiyuan 030001, China; xiangjieguo1980@yeah.net (X.G.); lqzhai3784@126.com (L.Z.); shijieru1980@sohu.com (J.S.); happy_zq1@163.com (Q.Z.); 2 Pathologic Department of People’s Hospital of Shanxi Province, 29 Shuang-ta Street, Taiyuan 030012, China; 3 Forensic Science Identification Center, No. 109 Hospital of Shanxi Province, 9 Tai-bao Street, Taiyuan 030006, China; rbxue_robin@163.com

**Keywords:** mast cell tryptase, pancreatic cancer, angiogenesis, angiopoietin-1

## Abstract

Pancreatic cancer is a highly lethal malignancy and one of the leading causes of cancer-related death. During the development and progression of cancer, tumor angiogenesis plays a crucial role. A great deal of evidence has revealed that human mast cells (MCs) contributed to tumor angiogenesis through releasing several pro-angiogenetic factors, among which tryptase is one of the most active. However, the role of mast cell tryptase (MCT) in human pancreatic cancer angiogenesis is still not well documented. In this study, we examined the MCT levels in serum from pancreatic cancer patients and evaluated the correlationship of the MCT level and tumor angiogenesis. In addition, the effect of MCT on endothelial cell proliferation and tube formation was investigated both *in vitro* and in nude mice bearing pancreatic tumor. It was found that MCT contributes to endothelial cell growth and tube formation via up-regulation of angiopoietin-1 expression. Moreover, using the MCT inhibitor nafamostat, tryptase-induced angiogenesis was obviously suppressed both *in vitro* and *in vivo*. Our findings suggest that MCT plays an important role in pancreatic cancer angiogenesis and tumor growth via activating the angiopoietin-1 pathway, and tryptase inhibitors may be evaluated as an effective anti-angiogenetic approach in pancreatic cancer therapy.

## 1. Introduction

Pancreatic cancer is one of the most common malignancies worldwide, in which pancreatic ductal adenocarcinoma (PDAC) is the most common type [1]. Despite remarkable research efforts by clinicians and cancer scientists, pancreatic cancer patients still have a low five-year overall survival rate of less than 5% [2], which is mainly due to difficulty in detecting pancreatic cancer at early stages, local aggression and rapid progression [3]. As there is still a less effective therapy method, it is urgent for us to improve our understanding of pancreatic cancer development and progression. A better understanding of the mechanism of pancreatic cancer pathogenesis will facilitate early detection and development of effective treatments for this deadly disease.

Angiogenesis plays a key role in tumor growth *in situ* and at a distance, affects multiple kinds of tumors including pancreatic cancer, and is a well-established anti-tumor target [4,5,6]. Recent studies indicated that mast cells (MCs) are involved in the regulation of extracellular matrix degradation, immune response and tumor angiogenesis via releasing numerous bioactive substances such as pro-angiogenic factors, including vascular fibroblast growth factor-2 (FGF-2), platelet-derived growth factor (PDGF), vascular endothelial growth factor (VEGF), interleukin-6 (IL-6) and some non-classical pro-angiogenic factors, for example tryptase [7,8]. Kankkunen *et al.* found that there were significantly more tryptase-containing MCs in malignant breast carcinomas compared to benign lesions, suggesting that MC density is correlated with the extent of tumor growth by promoting angiogenesis [9,10]. Meanwhile, MCs tryptase (MCT) had been found to generate a similar effect as VEGF to stimulate angiogenesis [11]. It was also reported that in pancreatic cancer, mast cells infiltrated to tumors were associated with a worse prognosis [12]. However, the correlations between MCT and angiogenesis in pancreatic cancer and the underlying mechanism are still not clear. Therefore, exploring whether the MCT is responsible for tumor angiogenesis could provide new diagnostic markers and therapeutic targets for cancers.

Tryptase is preformed active serine protease that represents approximate 50% of the total proteins in the MC secretory granule [13], whose angiogenic role has been determined [14,15]. In particular, tryptase represents one of the most powerful angiogenic mediators released by human MCs, and it is involved in angiogenesis after being released from activated MC granules [16]. Additionally, Blair *et al.* reported that tryptase is a mitogen for human dermal microvascular endothelial cells and causes a significant augmentation of capillary growth, which could be suppressed by specific tryptase inhibitors [15,17]. However, the biological function of MCT in angiogenesis and tumor growth in pancreatic cancer is still not well documented. In this study, we detected the MCT level in serum and tumor tissues from pancreatic cancer patients and analyzed the correlationship between the MCT level and angiogenetic index. Furthermore, the role of MCT and its inhibitor in human umbilical vein endothelial cells (HUVEC) proliferation, tube formation ability and underlying pathways were also investigated both *in vitro* and *in vivo*. Our findings indicate that MCT contributes pancreatic cancer angiogenesis and tumor growth, and its specific inhibitors may be potential agents for pancreatic cancer.

## 2. Results

### 2.1. Increased MCT Level in Pancreatic Cancer Patients Correlates with Tumor Angiogenesis

**Figure 1:**
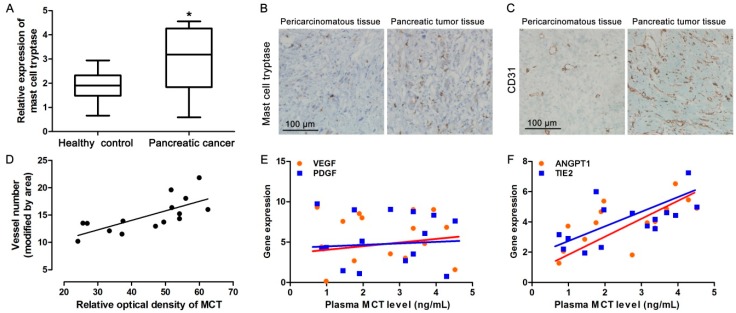
Correlationship of MCT and pro-angiogenic markers in pancreatic cancer patients. (**A**) Relative expression level of MCT in serum from pancreatic cancer patients (*n* = 15) and healthy people (*n* = 10) detected by ELISA. Wilcoxon Signed Ranks Test was used to compare significant difference, * *p* < 0.05; (**B**) IHC staining using MCT antibody in pericarcinomatous and tumor tissues from pancreatic cancer patients, representative image, scale bar = 100 μm; (**C**) IHC staining using CD31 antibody in pericarcinomatous and tumor tissues from pancreatic cancer patients, representative image, scale bar = 100 μm; (**D**) The relationship between MCT expression levels and microvascular density in pancreatic cancer tissues were analyzed by using Pearson’s (*r*) analysis, *r^2^* = 0.52, *p* < 0.01, *n* = 15; (**E**) and (**F**) The mRNA expression levels of VEGF, PDGF, ANGPT1 and TIE2 were detected by qPCR, GAPDH was used as internal reference. The relationship between serum MCT levels and VEGF, PDGF, ANGPT1 and TIE2 mRNA expression levels in pancreatic cancer tissues were analyzed by using Pearson’s (r) analysis, *n* = 15. There was less relationship between serum MCT levels and VEGF or PDGF. *r^2^* = 0.41, *p* < 0.05 in ANGPT1, *r^2^* = 0.38, *p* < 0.05 in TIE2, respectively.

To investigate whether MCT was involved in pancreatic cancer development, we firstly examined the expression level of MCT in serum from pancreatic cancer patients and healthy people. As is shown in Figure 1A, the level of serum tryptase is significantly higher in pancreatic cancer patients than in the control. Meanwhile, immunohistochemical (IHC) staining for MCT showed that the MCT level was also up-regulated in pancreatic cancer tissues compared to that in matched pericarcinomatous tissues (Figure 1B). Additionally, IHC staining of a specific vascular endothelial cell marker, CD31, indicated that the microvascular density in pancreatic cancer tissues was higher than that in pericarcinomatous tissues (Figure 1C). Using correlation analysis, it was found that the increased MCT levels were correlated with higher microvascular density in pancreatic cancer patients (Figure 1D). We further detected the expression of some classical pro-angiogenic factors. The mRNA expression levels of VEGF, PDGF, angiopoietin-1 (ANGPT1) and TIE2 in tumor tissues were analyzed by qPCR. Interestingly, the serum MCT level was less correlated with two important pro-angiogenic factors, VEGF or PDGF, in pancreatic cancer patients (Figure 1E), but highly related to ANGPT1 and TIE2 expression levels (Figure 1F). These data confirmed that increased MCT could be involved in pancreatic cancer development through affecting angiogenesis.

### 2.2. MCT Promoted Proliferation and Vascularization in HUVEC

**Figure 2:**
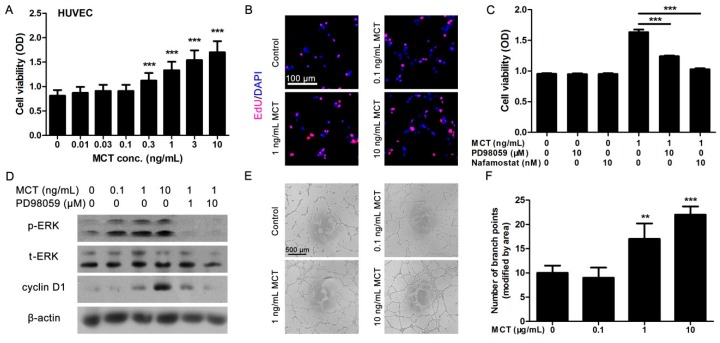
MCT promotes HUVEC proliferation and tube formation. (**A**) After HUVEC were treated with different doses of MCT for 24 h, CCK8 assays were used to determine the cell viability, *** *p* < 0.001, *n* = 6; (**B**) After HUVEC were treated with different doses of MCT for 24 h, EdU assays were used to determine cell proliferation. Red fluorescence indicate EdU-positive cells, DAPI dye stained all nuclei. Scale bar = 100 μm and refer to all panels; (**C**) After HUVEC were treated with MCT and/or PD98095 or nafamostat for 24 h, cell viability was measured by CCK8 assay. *** *p* < 0.001, *n* = 6; (**D**) After HUVEC were treated with MCT and/or PD98095 for 24 h, the cells were lysed and Western blot assays were used to detect the protein levels of pERK-, ERK-, and cyclin D1–treated HUVEC. The β-actin was internal reference, all blots were repeated for at least three times; (**E**) Tube formation assays were performed in HUVEC cells in the presence of different concentration of MCT. Scale bar = 100 μm and refer to all panels; (**F**) The number of branch points in tube formation was analyzed, 15 random images in each group were selected for analysis, ** *p* < 0.01, *** *p* < 0.001. All statistical difference were analyzed by ANOVA and Student-Newman-Keuls tests.

To further assess the potential role of MCT in the angiogenetic process, the effects of MCT on the proliferation and tube formation ability of HUVEC were investigated. HUVEC were incubated with different concentrations of human recombined lung MCT for 24 h, then CCK8 assay was performed to determine the cell viability. It showed that MCT could significantly increase the cell viability of HUVEC (at the concentrations >0.3 ng/mL) in a dose-dependent manner (Figure 2A). EdU staining further confirmed that MCT could promote HUVEC proliferation (Figure 2B). As predicted, co-treated cells with MCT inhibitor nafamostat (10 nM) could partly reverse the proliferative effect of MCT on HUVEC (Figure 2C). As the mitogen-activated protein kinase (MAPK) pathway is known to participate in cell proliferation, we used PD98059, an inhibitor of extracellular signal-regulated kinase (ERK) phosphorylation, to investigate whether the MAPK pathway was involved in MCT-induced HUVEC proliferation. It was found that like nafamostat, PD98059 also suppressed the proliferative effect of MCT (Figure 2C). Moreover, Western blot experiments showed that the phosphorylated-ERK (p-ERK) protein levels were increased in HUVEC cells after stimulation with 1 and 10 ng/mL MCT for 24 h, while PD98095 blocked the promoting effect of MCT on ERK phosphorylation (Figure 2D). As a result, cyclin D1, a cell cycle–dependent protein strongly indicating cell proliferation, was found to be increased in MCT-stimulated HUVEC, while PD98059 dose-dependently suppressed this effect (Figure 2D).

In addition, tube formation assays indicated that MCT at a dose of 1 and 10 ng/mL significantly increased the tube formation ability of HUVEC cells (Figure 2E,F), which suggested that MCT could not only promote endothelial cell growth but also activate vascularization, thereby potentially promoting pancreatic cancer growth via angiogenesis.

### 2.3. ANGPT1 was Involved in MCT-Induced Promotion of Angiogenesis

**Figure 3:**
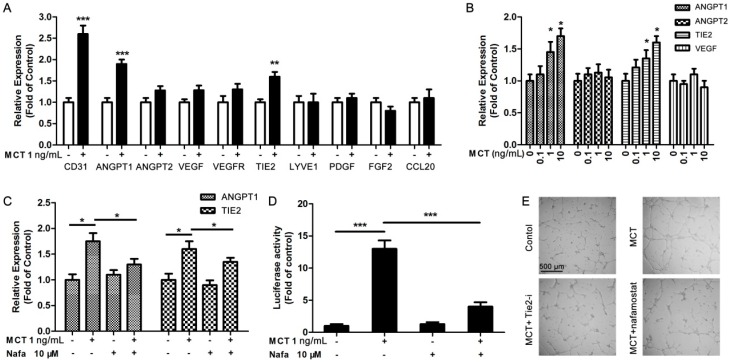
MCT activated HUVEC function via up-regulation of ANGPT1 pathway. (**A**) qPCR was used to detect the expression levels of angiogenesis-related gene expression in HUVEC treated with 1 ng/mL MCT for 2 h; ** *p* < 0.01, *** *p* < 0.001 compared to control, *n* = 3; (**B**) ANGPT1, ANGPT2, VEGF and TIE2 expression levels were further detected by qPCR in HUVEC cells after 2 h treatment with dose-dependent MCT. * *p* < 0.05 compared to control, *n* = 3; (**C**) After HUVEC were treated with MCT or along with nafamostat (Nafa) for 2 h, ANGPT1 and TIE2 expression levels were detected by qPCR. * *p* < 0.05 as indicated, *n* = 3; (**D**) The activity of luciferase reporter containing ANGPT1 promoter region was examined after treatment with MCT or combined with nafamostat for 2 h, *** *p* < 0.001, *n* = 3. All statistical difference were analyzed by ANOVA and Student-Newman-Keuls tests; (**E**) Tube formation assays were performed to determine the tube formation ability of 1 ng/mL MCT only, 1 ng/mL MCT and 10 μM Tie2 inhibitor or 10 μM nafamostat co-treated HUVEC. Scale bar = 500 μm and refer to all panels.

To date, the underlying targets or pathways involved in MCT-induced promotion of angiogenesis in pancreatic cancer cells are still unknown. We detected the angiogenesis-related gene expression by using qPCR assay in HUVEC after 2 h treatment with MCT for the first time. The results showed that among those angiogenesis-related genes, ANGPT1 and TIE2 expression levels were significantly increased by 1 ng/mL MCT stimulation, while the positive control CD31 expression was also increased (Figure 3A). We further treated HUVEC with different concentrations of MCT, and confirmed that MCT could increase the ANGPT1 and TIE2 expression in a dose-dependent manner (significantly when the concentration was higher than 1 ng/mL). However, there was less difference in ANGPT2 and VEGF expression under MCT stimulation (Figure 3B). Importantly, in co-treated HUVEC with MCT and its inhibitor nafamostat, the promoting effect of MCT on ANGPT1 and TIE2 expression was obviously blocked (Figure 3C), further proving that MCT could affect the ANGPT1/TIE2 pathway. Luciferase reporter assays showed that MCT could increase the luciferase activity that contains the ANGPT1 promoter region, but along with nafamostat treatment, this effect was reversed (Figure 3D). Finally, tube formation assays showed that TIE2 inhibitor ab141270 could partly block MCT-induced tube formation as well as the MCT inhibitor nafamostat (Figure 3E). These findings suggested that MCT could up-regulate ANGPT1 expression to activate ANGPT1/TIE2 pathway, which could be strongly involved in MCT-induced endothelial cell proliferation and angiogenesis.

### 2.4. MCT Promotes Pancreatic Cancer Cells Tumorigenesis and Angiogenesis *in Vivo*

**Figure 4:**
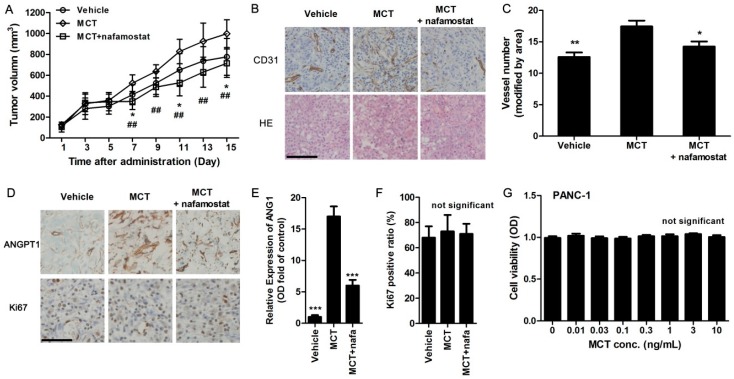
MCT promotes pancreatic cancer cells tumorigenesis via increasing angiogenesis *in vivo*. (**A**) Nude mice bearing pancreatic tumor were subcutaneously injected with MCT (200 ng/kg) or MCT along with nafamostat (10 mg/kg) daily, while vehicle mice received saline. The tumor volume was measured every two days after tumor formed with injection of PANC-1 cells. * *p* < 0.05 indicated MCT-treated group and vehicle group, ^##^ *p* < 0.01 indicated MCT-treated group and nafamostat co-treated group, *n* = 10; (**B**) After experiments, tumor tissues were removed and embedded for paraffin sections. IHC staining for CD31 and Hematoxylin-Eosin (HE) staining were performed, scale bar = 100 μm and refer to all panels; (**C**) The microvascular density in tumor tissues was calculated as CD31-positive vessel number (random 10 images, adjusted by tumor areas), * *p* < 0.05, ** *p* < 0.01 compared to MCT-treated group; (**D**) IHC staining for ANGPT1 and Ki67, nuclei were stained with hematoxylin, scale bar = 100 μm and refer to all panels; (**E**) Optical density (OD) was measured from 10 random images of ANGPT1 staining in each animal, *** *p* < 0.001 compared to MCT-treated group, *n* = 10; (**F**) Ki67-positive ratio was calculated as Ki67-positive nuclei number divided by total hematoxylin-stained nuclei, *n* = 10; (**G**) After PANC-1 cells were treated with different concentration of MCT for 24 h, CCK8 assays were used to determine the cell viability, *n* = 6. All statistical difference were analyzed by ANOVA and Student-Newman-Keuls tests.

To explore whether MCT could affect pancreatic cancer cell tumorigenesis *in vivo*, PANC-1 pancreatic cancer cells were inoculated into female nude mice. Once the tumors formed, the mice were subcutaneously injected with vehicle, human MCT (200 ng/kg) or co-injected with MCT with nafamostat (10 mg/kg). The tumor volume was examined every two days. Fifteen days after administration, the volume of the tumors formed in the MCT-treated group were substantially larger than those in the control group, while the volume of the tumors formed in combined administration with nafamostat was substantially smaller than those in the MCT and control groups (Figure 4A). Moreover, the results of IHC staining by using anti-CD31 antibodies indicated that the vessel number in tumor tissues collected from the MCT group is higher than that in the control group, while the vessel number in MCT combined with nafamostat group is decreased compared to the other two groups (Figure 4B,C). Meanwhile, the expression of ANGPT1 in tumor tissues and Ki67-positive proliferative cells was also evaluated by IHC staining using ANGPT1 or Ki67 antibody. In accordance with the result of CD31 staining, the ANGPT1 expression level was obviously increased in MCT-treated mice compared with vehicle, while co-treatment with nafamostat reversed the promoting effect of MCT on tumor growth (Figure 4D,E). Thus, combined with the results in pancreatic cancer patients, angiogenesis could be correlated with the MCT-stimulated increasing in tumor size. However, the Ki67-postitive cell ratio had no significant difference among all three groups (Figure 4D,F), which suggested that promoting tumor cell proliferation was less involved in MCT-improved pancreatic tumor growth. The effect of MCT on the cell viability of PANC-1 pancreatic cancer cells was performed to confirm this conclusion, and it was also proved that MCT did not affect PANC-1 cell growth *in vitro* (Figure 4G).

## 3. Discussion

Human MCs are a rich reservoir of neutral proteases including dipeptidylpeptidase, cathepsin G, carboxypeptidase A3, chymase and tryptase, which were packed in large amounts in MC granules [18]. Among these neutral proteases, tryptase is a powerful angiogenic mediator after its release from activated human MC granules, which may activate angiogenic activity through multiple mechanisms [19,20,21]. Moreover, lots of studies have demonstrated that tryptase-positive MCs increase in number and vascularization increases in a linear fashion in several types of solid tumors including multiple myeloma [22], melanoma [23], endometrial carcinoma [24], breast cancer [25], colorectal cancer [26], gastric cancer [27] and lung cancer [28]. For example, Girolamo *et al.* reported that there is a significant correlation between MCs positive for tryptase and the area occupied by MCs positive for tryptase, microvascular density, or endothelial area in a series of 88 primary female breast cancers [29]. These findings all indicate that MCT plays a critical role in cancer progression.

In this study, we also found significantly increased MCT levels in serum and tumor tissue samples from pancreatic cancer patients, and the expression level of MCT was correlated with microvascular density in pancreatic cancer tissues. Therefore, we further detected the expression level of some classical angiogenic factors such as VEGF, PDGF, ANGPT1 and TIE2 [30]. Interestingly, although ANGPT1 and its receptor TIE2 showed high correlationship with the serum MCT level in pancreatic patients, VEGF and PDGF revealed less correlation. Thus, a possible signal transduction pathway, ANGPT1/TIE2 could participate in MCT-stimulated tumor angiogenesis.

Therefore, we evaluated the effect of MCT on angiogenesis using an endothelial cell line, HUVEC. The results showed that human MCT could promote HUVEC cells’ proliferation and tube formation ability. Using MCT inhibitor nafamostat, the effect of MCT on cell proliferation markers, such as the increased EdU-positive cell number, cell viability and expression of cell cycle protein cyclin D1, was specifically suppressed. Similar results were also proved in the tube formation assay. To further investigate the effect of MCT on pancreatic cancer, we established nude mice bearing pancreatic tumor. Our *in vivo* experiments revealed that MCT could promote pancreatic cancer cell tumorigenesis, and MCT-treated mice generated more vessel numbers in tumor tissue than the vehicle. This data was in consistent with the results that the MCT level was correlated to the vessel number in pancreatic cancer patients. We also investigated the expression of ANGPT1 in MCT-treated mice. Similar to clinical specimens, the MCT-treated nude mice bearing pancreatic tumor had higher expression levels of ANGPT1, while nafamostat co-injection partly suppressed the effect of MCT, which added the speculation that MCT regulated ANGPT1 expression to affect angiogenesis in pancreatic cancer. However, whether MCT directly affects pancreatic tumor cell growth still must be clarified. Ki67 staining showed a similar tumor cell growth level between MCT-treated mice and vehicle, while the *in vitro* cell viability assay also showed that MCT did not affect PANC-1 pancreatic cancer cell line growth, strongly suggesting that MCT affected tumor cell growth less but mainly promoted angiogenesis in pancreatic cancer.

In this study, we first reported that ANGPT1 may be an important underlying regulator that is involved in MCT-induced angiogenesis in pancreatic cancer. In endothelial cells, ANGPT1 activates its receptor TIE2, thereby phosphorylating the downstream MAPK pathway [31]. MAPK signaling pathways are well-known regulators of cell survival, proliferation and migration, which contribute to angiogenesis [32]. Meanwhile, it was reported that MCT could promote colon carcinoma cell growth via activating the MAPK pathway [33]. We also found similar results that the phosphorylation level of one MAPK, extracellular signal-regulated kinase (ERK), could be dose-dependently increased by MCT. Using MAP kinase inhibitor PD98059 to inhibit the phosphorylation of ERK, MCT-induced cell proliferation was suppressed, suggesting that the activation of the MAP kinase pathway was at least partly involved in the proliferative effect of MCT. Furthermore, we found that MCT stimulation could specifically up-regulate the expression of ANGPT1 and TIE2 in HUVEC. Using luciferase assay, it was found that MCT could increase the gene transcriptional expression of ANGPT1, finally promoting endothelial cell growth and angiogenesis. Furthermore, both the Tie-2 inhibitor and MCT inhibitor could abolish the effect of MCT. Taking together the *in vitro*, *in vivo* and clinical data, it suggests a possible signal transduction pathway: MCT-ANGPT1-TIE2-MAPK-endothelial cell proliferation and angiogenesis. In combination with previous reports, these findings suggest that the complex interplay between MCs and tumor angiogenesis may lead to consideration of the use of angiogenesis inhibitors, which specifically target the tryptase angiogenic activity, such as nafamostat, an inhibitor of trypsin-like serine proteases, for cancer therapy [34,35].

## 4. Materials and Methods

### 4.1. Serum Analysis and IHC Assay

ELISA assay was performed to determine serum MCT level in pancreatic cancer patients and healthy adults using MCT antibody (SantaCruz Biotechnology, SantaCruz, CA, USA) and microtiter plate (Coster, CA, USA). To further evaluate the association of mast cell tryptase (MCT) level and microvascular density (MVD), 4 μm serial sections of formalin-fixed and paraffin-embedded biopsy pancreatic cancer tissues were deparaffinized. Then, sections were microwaved for 10 min for antigen retrieval, and endogenous peroxidase activity was blocked with 3% hydrogen peroxide solution. Next, slides were incubated with human-specific monoclonal antibodies anti-tryptase (SantaCruz Biotechnology) diluted 1:100 for 1 h and anti-CD31 (Cell Signaling Technology, Beverly, MA, USA) diluted 1:100 for 12 h at room temperature. Biotinylated anti-Rabbit IgG (1:200; Sigma Aldrich, St. Louis., MO, USA) was used as secondary antibody. Nuclear were stained by hematoxylin (Beyotime, Shanghai, China). Finally, sections were mounted onto slides using Gel Mount Aqueous Mounting Medium (Sigma Aldrich) and captured with an Olympus BX51 microscope (Olympus, Tokyo, Japan). Images were analyzed by Image J software (the National Institutes of Health, Bethesda, MD, USA). For morphological analysis, pericarcinomatous tissues (more than 2 cm from nidus) were reserved. All experiments were permitted by the Ethic Committee of Shanxi Medical University (2011004, January 2011).

### 4.2. qPCR Assay

The mRNA expression of ANGPT1, TIE2, VEGF and PDGF were determined by real-time PCR as previous described with minor modification [36]. Briefly, tumor tissues from pancreatic cancer patient was used to isolate mRNA by Trizol (Takara, Tokyo, Japan). Primers using for each mRNA were in Table S1.

### 4.3. Cell Culture

HUVEC cells and pancreatic cancer cell line PANC-1 cell were purchased from the Institute of Biochemistry and Cell Biology of the Chinese Academy of Sciences (Shanghai, China). HUVEC cells were cultured in RPMI 1640; PANC-1cells were cultured in DMEM (GIBCO-BRL) medium supplemented with 10% fetal bovine serum (FBS), 100 U/mL penicillin and 100 mg/mL streptomycin (Invitrogen, Carlsbad, CA, USA) at 37 °C/5% CO_2_.

### 4.4. Cell Proliferation Assays

Cell proliferation was analyzed using a Cell Proliferation Reagent Kit I (CCK8) (Dojindo, Tokyo, Japan). The cells were treated with tryptase (0, 0.01, 0.03, 0.1, 0.3, 1, 3 and 10 ng/mL) or along with PD98059 and nafamostat (10 μM). Cell proliferation was assessed 24 or 48 h after different treatments following the manufacturer’s protocol. All experiments were performed in quadruplicate. Meanwhile, to further evaluate cell proliferation, EdU assay was used to mark replicated cells following its protocol (Invitrogen, Carlsbad, CA, USA).

### 4.5. Tube Formation Assay

For tube formation assays, cell culture inserts were coated with growth factor reduced matrigel (BD Biosciences, Oxford, UK). A total of 2.5 × 10^4^ HUVEC cells re-suspended in media containing 1% FBS were plated into the upper layer of the chamber previously coated with matrigel. The cells were incubated at 37 °C with 5% CO_2_ for 24 h, then the cells in each well were image captured under a microscope. Each experiment was performed in duplicate.

### 4.6. Western Blotting Analysis

HUVEC cells were lysed using RIPA protein extraction reagent (Beyotime) with protease inhibitor cocktail and phenylmethylsulfonyl fluoride (Roche, Indianapolis, IN, USA). The total protein concentration was determined using the Bio-Rad protein assay kit (Bio-Rad, Hercules, CA, USA). Protein extracts (40 µg) were separated by 10% sodium dodecyl sulfate-polyacrylamide gel electrophoresis (SDS-PAGE), then transferred to PVDF membranes and incubated with antibodies. Autoradiograms were quantified by densitometry using Quantity One software (Bio-Rad), with β-actin used as a control. Antibodies (1:1000 dilution) against ERK, pERK, Cyclin D1 and β-actin were purchased from Cell Signaling Technology.

### 4.7. Tumor Formation Assay in a Nude Mouse Model

Four-week-old female athymic BALB/c nude mice were maintained under specific pathogen-free conditions and manipulated according to protocols. PANC-1 cells were harvested, washed with PBS, and re-suspended at a concentration of 1 × 10^7^ cells/100 μL. A volume of 100 µL of suspended cells was subcutaneously injected into the posterior flank of each mouse. The mice were subcutaneous injected with 200 ng/kg MCT or co-injected with 10 mg/kg nafamostat daily. Tumor growth was examined every two days, and tumor volumes were calculated using the equation *V* = 0.5 × *D* × *d*^2^ (*V*, volume; *D*, longitudinal diameter; *d*, latitudinal diameter). At 15 days post MCT or nafamostat treatment, mice were euthanized, tumor tissues were removed and embedded for paraffin sections. IHC assay was performed using anti-CD31, -ANGPT1, -Ki67 antibodies (SantaCruz). The MVD, ANGPT1 expression and Ki67-positive ratio in each group was examined by Image J software. The protocol was approved by the Ethic Committee of Shanxi Medical University (No. 2011004).

### 4.8. Statistical Analysis

All data are presented as means ± SD. ANOVA and Student-Newman-Keuls tests were used for multiple comparisons and a value of *p* < 0.05 was considered significant. Difference in serum MCT level between healthy people and pancreatic cancer patients was calculated using Wilcoxon Signed Ranks Test. Correlations between MCT and MVD were calculated using Pearson’s (r) analysis. All statistical analysis were performed with the SPSS 17.0 statistical software package (IBM, Armonk, NY, USA).

## 5. Conclusions

In summary, we demonstrate that the levels of MCT are increased in pancreatic cancer patient serum and tissues, and a strong correlationship between the MCT level and angiogenesis was shown. The evidence that the ANGPT1/TIE2 pathway is involved in this process was first provided in this study. Moreover, MCT could promote pancreatic cancer cell tumorigenesis *in vivo* through promoting angiogenesis but could not directly affect tumor proliferation; this effect could be specifically blocked by tryptase inhibitor nafamostat. Therefore, MCT inhibitors such as nafamostat and gabexate mesilate may be proposed as anti-angiogenic agents in combination with chemotherapy in the treatment for human pancreatic cancer.

## Supplementary Materials

Supplementary materials can be found at http://www.mdpi.com/1422-0067/17/6/834/s1.
