# Supplementary material for: Mast Cell Tryptase Contributes to Pancreatic Cancer Growth through Promoting Angiogenesis via Activation of Angiopoietin-1
Source: Int J Mol Sci. 2016 May 27;17(6):834. doi: 10.3390/ijms17060834 (PMC4926368; doi:10.3390/ijms17060834)
Supplement: Supplementary file 1 [file ijms-17-00834-s001.pdf]

Supplementary Materials: Mast Cell Tryptase Contributes to Pancreatic Cancer Growth through Promoting Angiogenesis via Activation of Angiopoietin-1

Xiangjie Guo, Liqin Zhai, Ruobing Xue, Jieru Shi, Qiang Zeng and Cairong Gao

Table S1. Primers used.

Gene Name	Forward (5′–3′)	Reverse (5′–3′)
ANGPT1	AGCGCCGAAGTCCAGAAAAC	TACTCTCACGACAGTTGCCAT
ANGPT2	AACTTTCGGAAGAGCATGGAC	CGAGTCATCGTATTCGAGCGG
TIE2	CGAGTTCGAGGAGAGGCAATC	TCAGGTACTTCATGCCGGG
LYVE1	AATTTCACAGAAGCTAAGGAGGC	TCAAGGCTGTTTCAACTTGGTC
PDGF	TGATGCCGAGGAACTATTCATCT	TTTCTTCTCGTGCAGTGTCAC
VEGF	GGCTGGCAACATAACAGAGAA	TCCTTTCCTTAGCTGACACTTGT
VEGFR	AAGGCCGTGTCATCATTTCCAGAC	ACCACTTGATTGTAGGTCGAGGGA
CD31	CCAAGGTGGGATCGTGAGG	TCGGAAGGATAAAACGCGGTC
FGF2	ATGGCTCCCTTAGCCGAAGT	AGGAAATGCGAACCCACCTG
CCL20	AATCAGAAGCAGCAAGCAACT	TTTTACTGAGGAGACGCACAA
GAPDH	CGGAGTCAACGGATTTGGTCGTAT	AGCCTTCTCCATGGTGGTGAAGAC
